# Factors affecting satisfaction with cancer information provided through the social networking services of the National Cancer Information Center in Korea

**DOI:** 10.4178/epih.e2017057

**Published:** 2017-12-11

**Authors:** Su Yeon Kye, Min Hee Lee, Jisu Yoo, Kyung Hee Oh, Jae Kwan Jun

**Affiliations:** Cancer Information and Education Branch, National Cancer Control Institute, National Cancer Center, Goyang, Korea

**Keywords:** Facebook, National Cancer Information Center, Satisfaction, Social network service, Twitter

## Abstract

**OBJECTIVES:**

This study aimed to identify the characteristics of social networking service (SNS) users and to assess the relationship of those factors to user satisfaction with the SNSs of the National Cancer Information Center (NCIC) in South Korea.

**METHODS:**

A Web-based survey was completed by 1,670 users of the NCIC SNSs, who provided data on the sources they consulted for information about cancer, determinants of SNS use, sources of NCIC SNS awareness, the time of day they used the SNS, and their satisfaction level.

**RESULTS:**

Facebook users mainly became aware of the NCIC SNSs through an acquaintance’s recommendation, while Twitter was accessed through other SNSs or blogs. Users in their 30s were less satisfied with the NCIC SNSs than those in their teens and 20s. Browsing for cancer-related information on the Internet, prioritizing information quality, and engaging in active use were related to a high level of satisfaction with the NCIC SNSs. Individuals who were exposed to the NCIC SNSs through other SNSs or printed materials were less satisfied with their experience of the NCIC SNSs than those who received a recommendation from an acquaintance.

**CONCLUSIONS:**

These findings may act as a catalyst to guide public health agencies to enhance their use of SNSs.

## INTRODUCTION

First introduced in 2004, the concept of Web 2.0 has dramatically changed the way people interact with information online, moving from passive consumption to active creation and sharing of content [1]. The construction of social networking services (SNSs) that enabled interactive communication led to an outpouring of information from people based on their own knowledge and experiences, increasing their sense of belonging and their desire for self-expression, and leading in turn to the development of various new SNSs and a huge increase in the number of users [2-4]. Presently, Facebook is the most visited SNS platform in the world, followed by YouTube and Twitter [5]. Launched in February 2004, Facebook had more than 2 billion monthly active users as of June 2017, compared to 328 million monthly active users of Twitter [6,7].

A growing number of public healthcare organizations have realized the advantages of the opportunities offered by SNS for communication and social interactions, not only to highlight their services but also to promote loyalty [8]. In the US, about 60% of state health departments reported using at least 1 SNS account in 2011 [9]. In Korea, the major public health organizations, such as the Ministry of Health and Welfare, the Korea Centers for Disease Control and Prevention, and the National Health Insurance Service, have adopted and used SNSs to deliver health information and to improve national and individual health.

The National Cancer Information Center (NCIC) was launched in Korea as a national cancer information service in 2005 to provide evidence-based cancer information via phone calls, through the NCIC website, and through publications. Since 2011, the NCIC has been using SNS platforms such as Twitter and Facebook to disseminate comprehensive information ranging from cancer prevention to diagnosis, treatment, and palliative care. As of October 2016, the NCIC had 29,024 Twitter followers and 18,137 Facebook friends [10,11].

User information satisfaction is defined as the extent to which users believe the information system that is available to them meets their information requirements [12]. Satisfaction appraisal is generally an important mediator of behavior that links communication, affect, cognitive beliefs, attitudes, and behavioral intentions to behavior modification [13,14]. Users with high levels of satisfaction show higher levels of behavioral intentions and less complaining behavior [13].

Although a vast amount of health information has been developed, revised, distributed, and consumed through SNSs, research into the factors affecting the satisfaction of SNS users has not been common. Early research examining SNSs has focused predominantly on the characteristics of these networking sites’ providers and users, user-generated content analysis, the current status of information sharing, and the impact of these SNSs on health promotion and disease treatment [15-20]. The purpose of the current study was to extend the existing research examining the characteristics of SNS users and to assess the relationship of their characteristics with user satisfaction with the NCIC SNSs in Korea. Based on this analysis, we present some implications for how public health agencies can operate SNSs more efficiently by increasing user satisfaction, with the ultimate goal of inducing users to improve their health practices.

## MATERIALS AND METHODS

### Design and respondents

A survey measuring users’ level of satisfaction with the NCIC SNSs was conducted 4 times between 2012 and 2014. The participants in this cross-sectional study included NCIC Twitter followers and Facebook friends who had signed up for the pages 3-5 months before the survey period to maximize the response. In 2012 and 2014, 1 survey was conducted, while 2 surveys were conducted in 2013. Nearly 1,500 individuals were sent messages inviting them to participate in each survey based on random sampling. The final analysis included 1,670 users (response rate, 27.8%). After completing the survey, some respondents were randomly chosen to receive small gifts. Informed consent was obtained from all individual participants included in the study. The research protocol was approved by the National Cancer Center institutional review board in Korea (NCC2016-0021).

### Measures

#### Overall satisfaction level

To measure users’ satisfaction levels, participants were asked in general terms about whether they were satisfied with the NCIC SNS, based on previous research into the use of a single item [21,22]. Scores ranged from 1 (definitely no) to 5 (definitely yes), with higher scores indicating a higher level of satisfaction with the services.

#### Cancer information sources

Participants were asked where they sought information about cancer, and could choose among the following 9 options: medical professionals, friends, family, cancer patients, books, newspapers, the Internet, TV, and booklets. Friends, family, and cancer patients were combined as personal sources, books and booklets were categorized as books, and newspapers and TV were considered to be mass media.

#### Determinants for using social networking service

Participants were asked why they considered using the SNS, and could choose among the following 8 options: a recommendation from an acquaintance, ease of use, high accessibility, information diversity, high reliability, usefulness, timeliness, and relevance. Ease of use and high accessibility were combined into the category of system quality; information diversity, high reliability, usefulness, and relevance were grouped as information quality, and a recommendation from an acquaintance and timeliness were categorized as communication quality [23].

#### National Cancer Information Center social networking service use-related variables

Participants were asked how actively they used the NCIC SNSs. For Twitter, the level of participation was classified into 4 categories: none; only following; following and reading; and following, reading, and replying or re-tweeting. Only following and following and reading were considered as passive, while following, reading, and replying or re-tweeting was regarded as active. On Facebook, the level of participation was divided into 5 categories: none; adding as a friend; liking; liking and reading; and liking, reading, and commenting or sharing. Adding as a friend, liking, and liking and reading were considered passive, while liking, reading, and commenting or sharing was regarded as active. The source of NCIC SNS awareness was assessed by the participants’ responses to predefined categories, such as a recommendation from an acquaintance, other SNSs, online searching, a website/blog of NCIC, online events hosted by NCIC, and printed materials. Participants were asked what time of the day they usually used the NCIC SNSs: late night/early morning (1-5 a.m.), commuting hours (6-8 a.m.), morning (9 a.m. to 12 p.m.), afternoon (1-6 p.m.), evening (7-9 p.m.), and night (10 p.m. to 12 a.m.).

#### Socio-demographic characteristics

These variables included gender, age, occupation, and cancer patient status. Occupational categories were classified as health/medical, management/professional, office, service/sales, and outdoor/homemaker. Participants were asked whether they or any of their family members were cancer patients.

### Analysis

The chi-square test was used to examine the relationships of cancer information sources, determinants of SNS use, sources of NCIC SNS awareness, time of day of SNS use, and socio-demographic characteristics with the use of Twitter and Facebook and engagement with NCIC content on Twitter and Facebook. Multivariate logistic analysis was subsequently performed to determine the adjusted odds ratios (aORs) for the use of Twitter and Facebook and engagement with NCIC content on Twitter and Facebook after adjusting for variables that achieved marginal significance (p< 0.1) in the univariate analysis. The t-test and analysis of variance were conducted to examine the relationships of cancer information sources, determinants of SNS use, sources of NCIC SNS awareness, the time of day of SNS use, the use of NCIC SNSs, and socio-demographic information with satisfaction levels. A binary linear regression analysis was subsequently performed to identify factors that were significantly related to satisfaction levels after adjusting for variables that achieved marginal significance (p< 0.1) in the univariate analysis. Data were analyzed using SPSS version 15.0 (SPSS Inc., Chicago, IL, USA).

## RESULTS

The respondents consisted of 1,670 Korean NCIC SNS users. Facebook was used more often than Twitter, and information quality was the most important factor for selecting the SNSs. The NCIC SNSs were mainly found through other SNSs following online searching. The mean satisfaction score was 3.95 out of a possible score of 5 (Table 1).

Table 2 shows the relationships between the characteristics of the users and SNS use. Older age was associated with Twitter use, while there was no significant difference for Facebook. People who sought information about cancer from the mass media or the Internet used SNSs more than those who did not, while those who sought information from personal contacts made less use of the NCIC SNSs. Seeking information about cancer from medical professionals was significantly related to more use of the NCIC Facebook page. Facebook was more commonly used by people who considered information quality and communication quality to be the most important factors for selecting an SNS, although considering system quality to be important was significantly associated with Twitter use. The NCIC Facebook page was often found through recommendations from an acquaintance, whereas other SNSs and the NCIC homepage or blog were significant mediators for users to find the NCIC Twitter account. People usually used the SNSs during the afternoon (1-6 p.m.) and at night (10 p.m. to 12 a.m.).

Respondents in their 30s were less likely to be satisfied with the NCIC SNSs than teens and users in their 20s. Individuals who sought information about cancer from the Internet were more likely to be satisfied with the services than those who did not. People who regarded information quality as the most important factor for selecting an SNS were more likely to be satisfied with the services. Active engagement with the NCIC SNSs was significantly related with higher levels of satisfaction. Users who were exposed to NCIC SNSs through other SNSs or printed materials were less satisfied with the NCIC SNSs than those who received a recommendation from an acquaintance (Table 3).

## DISCUSSION

This study examined the factors affecting respondents’ satisfaction with the NCIC SNSs. Users in their 30s were less satisfied with the NCIC SNSs than those in their teens and 20s. Seeking information about cancer from the Internet, considering information quality to be an important factor, and active engagement were related to higher levels of satisfaction with the NCIC SNSs. Respondents who were exposed to NCIC SNSs through other SNSs or printed materials were less satisfied with the NCIC SNSs than those who received a recommendation from an acquaintance.

A significant relationship was found between age and SNS use. People in their 30s and 40s were more likely to use Twitter than teenagers and those in their 20s, whereas no age difference was seen in Facebook use. In addition, Twitter was more popular among people over the age of 40 than the younger age group, although people in their 30s were more likely to use Facebook than their younger counterparts, suggesting that the age of SNS users might have been higher among Twitter users than Facebook users. Previous studies have revealed that, in 2010, people over the age of 40 preferred to use Twitter, while those in their 20s preferred Facebook [24]. In light of this finding, it seems reasonable to conclude that health communication efforts using Twitter will have an impact on a target population that is relatively older, while Facebook appears to be more effective for reaching younger people.

The choice of SNS varied by the determinant that people considered to be important. Twitter was relatively more popular among those who thought that system quality was the most important factor in using an SNS, while Facebook users placed more emphasis on information quality and active communication. These results are similar to those of a study in which Facebook was shown to be used to communicate more and to foster deeper relationships than Twitter [25]. This could be explained by the fact that Twitter has an upper limit on the number of characters, whereas Facebook provides space for a profile to introduce oneself and utilizes direct comments. Those who followed the NCIC Twitter account were less likely to seek information about cancer from personal contacts and more likely to do so from the mass media, while NCIC Facebook users were less likely to seek cancer information from the mass media.

The sources of SNS awareness differed according to the pattern of NCIC SNS use. The proportion of respondents indicating that someone recommended the NCIC Facebook page to them was larger than the corresponding proportion for Twitter. Regarding Twitter, individuals tended to begin using the NCIC SNS by reading NCIC SNS-related posts in other SNSs or on NCIC blogs. These findings indicate that Facebook’s interactive characteristics were associated with a greater importance of human networking, whereas Twitter showed stronger connectivity among users’ content. In this way, a recommendation from an acquaintance can play an important role in providing social support when people engage in health information-seeking behavior, which can lead to positive health behavior changes [26].

This study showed that satisfaction levels with the NCIC SNSs were associated with the determinants of SNS use, and those who regarded information quality as the most important factor for choosing a type of SNS were more satisfied with the NCIC SNSs than those who did not. This might mean that information credibility should be considered important, even in the context of SNSs. When consumers use a service, they form perceptions about its performance, assess it face to face their original expectations, form a certain level of satisfaction, and determine whether they will continue to use it [27]. Therefore, individuals and organizations require information quality for health-related knowledge building and delivery [28]. The level of engagement with NCIC SNSs was significantly related to satisfaction; those who used the SNSs more actively were more satisfied with the services. In light of this result, the development and implementation of SNS strategies should be based on the needs of users; promising steps would include improving the convenience of use and developing a more active attitude toward the use of information that eventually leads to behavioral changes [23]. Regarding the sources of NCIC SNS awareness, those who were exposed to NCIC SNSs through other SNSs or printed materials were less satisfied with the NCIC SNSs than those who were received a recommendation from an acquaintance, suggesting the importance of social support online as well as offline. This result is in accordance with a previous study revealing that health-related services recommended by others were associated with higher satisfaction levels than those that were not [29].

Nonetheless, some limitations of this study should be noted. First, the study population was restricted to a relatively small sample of NCIC SNS users, so the results are not generalizable. Second, these findings were based on self-reports of satisfaction assessed using a single item and, therefore, some of the responses may not be entirely reliable. Last, this was a cross-sectional study, so cause-and-effect relationships could not be confirmed. Nevertheless, most importantly, this study demonstrated a range of meaningful variables for improving satisfaction in the use of the SNSs of national public health agencies. These findings could be a catalyst for developing recommendations about how other public health agencies should build and implement their social media platforms. Healthcare providers should focus their efforts on improving user satisfaction by improving the quality of their content. It is also possible to improve users’ satisfaction by identifying their informational needs and communicating information accordingly, thereby enhancing the users’ engagement with the SNS. Additionally, the most important promotion strategy is to utilize word of mouth, which is the most important factor for improving satisfaction levels with other public relations activities. The present study suggests that other determinants that were not dealt with in this study, such as knowledge, beliefs, attitudes, skills, and affective or environmental factors, should be considered in future research. In addition, extending this analysis to include an evaluation of intentions and actual use would probably provide a basis for recommendations for a future research agenda. Furthermore, further analyses incorporating qualitative aspects could investigate the reasons for lower levels of satisfaction with particular SNSs.

Given the rapid changes in the communication era brought about by SNSs, it is important to develop a better understanding of patterns of SNS use and users’ satisfaction levels. The purpose of the present study was to investigate the relationship of SNS users’ characteristics with their satisfaction with NCIC SNSs. Users in their 30s were less satisfied with NCIC SNSs than those in their teens and 20s. Seeking information about cancer on the Internet was related to greater satisfaction with NCIC SNSs. People who regarded information quality as the most important factor in choosing a type of SNS were more satisfied with NCIC SNSs than were those who did not. Those who used SNSs more actively were more satisfied with NCIC SNSs. Regarding the sources of NCIC SNS awareness, individuals who were exposed to NCIC SNSs through other SNSs or printed materials were less satisfied with the NCIC SNSs than those who received a recommendation from an acquaintance. Understanding how satisfaction can be increased would be helpful for SNS practitioners to develop and improve their SNSs, as well as for other online information systems. To implement SNSs effectively, it is necessary to utilize different SNS platforms depending on the targeted age group, and maximizing information quality is the most powerful strategy for improving satisfaction. It is also crucial to try to foster word-of-mouth marketing—that is, marketing between consumers—to increase satisfaction. Efforts should be made to further explore subtle cognitive and emotional factors affecting satisfaction through qualitative and quantitative studies. Measuring the factors affecting user satisfaction with the NCIC SNSs can serve as a benchmark for how public health agencies can improve their use of SNSs and encourage more research into the effectiveness of SNS use in public health.

## Figures and Tables

**Table 1.** Characteristics of participants and SNS usage behavior

	n (%)
Total	1,670 (100.0)
Gender	
Man	683 (40.9)
Woman	987 (59.1)
Age (yr)	
10-29	530 (31.7)
30-39	711 (42.6)
40-49	303 (18.1)
≥50	126 (7.5)
Occupation	
Health/medical	306 (18.3)
Management/professional	807 (48.3)
Office	95 (5.7)
Service/sales	383 (22.9)
Outdoor/housewife	79 (4.7)
Cancer patient status	
General public	1,206 (72.2)
Cancer patients or family	464 (27.8)
Cancer information sources (multiple responses)	
Medical professionals	507 (30.4)
Personal	1,547 (92.6)
Books	325 (19.5)
Mass media	629 (37.7)
Internet	1,253 (75.0)
Use of SNS (multiple responses)	
Twitter	866 (51.9)
Facebook	1,316 (78.8)
Determinant of SNS use	
System quality	606 (36.3)
Information quality	689 (41.3)
Communication quality	375 (22.5)
Use of NCIC Twitter	
None	666 (39.9)
Passive	596 (35.7)
Active	339 (20.3)
Use of NCIC Facebook	
None	311 (18.6)
Passive	776 (46.5)
Active	514 (30.8)
Source of NCIC SNS awareness	
Recommendation from a acquaintance	167 (10.0)
Other SNSs	665 (39.8)
Online searching	292 (17.5)
Website/blog of the NCIC	181 (10.8)
Online events hosted by the NCIC	226 (13.5)
Printed materials	139 (8.3)
Time of day of SNS use (multiple responses)	
1-5 a.m.	58 (3.5)
6-8 a.m.	172 (10.3)
9 a.m. to 12 p.m.	768 (46.0)
1-6 p.m.	903 (54.1)
7-9 p.m.	683 (40.9)
10 p.m. to 12 a.m.	362 (21.7)
Satisfaction (mean, SE)	3.95 (0.78)

SNS, social networking service; NCIC, National Cancer Information Service; SE, standard error.

**Table 2.** Factors associated with SNS use

	Twitter	Facebook	NCIC Twitter		NCIC Facebook	
	Use vs. no use (n=1,670)^1^	Use vs. no use (n=1,670)^2^	Use vs. no use (n=1,601)^3^	Active vs. passive use (n=935)^4^	Use vs. no use (n=1,601)^5^	Active vs. passive use (n=1,290)^6^
Gender						
Men	1.00 (reference)	1.00 (reference)		1.00 (reference)		
Women	1.37 (1.10, 1.70)	1.08 (0.83, 1.39)		1.10 (0.81, 1.50)		
Age (yr)						
10-29	1.00 (reference)	1.00 (reference)	1.00 (reference)		1.00 (reference)	1.00 (reference)
30-39	1.49 (1.16, 1.90)	1.03 (0.76, 1.38)	1.21 (0.94, 1.56)		1.47 (1.09, 1.99)	1.16 (0.86, 1.56)
40-49	1.87 (1.38, 2.55)	0.72 (0.50, 1.04)	1.76 (1.28, 2.43)		0.98 (0.68, 1.40)	1.74 (1.20, 2.51)
≥50	1.48 (0.98, 2.23)	0.88 (0.54, 1.44)	1.91 (1.24, 2.96)		1.11 (0.68, 1.81)	0.84 (0.50, 1.40)
Occupation						
Health/medical	1.00 (reference)	1.00 (reference)	1.00 (reference)	1.00 (reference)		1.00 (reference)
Management/professional	0.57 (0.43, 0.75)	0.72 (0.51, 1.03)	1.21 (0.83, 1.77)	0.92 (0.59, 1.44)		1.28 (0.85, 1.92)
Office	0.90 (0.55, 1.47)	0.67 (0.38, 1.19)	1.79 (0.99, 3.23)	0.70 (0.32, 1.52)		1.22 (0.63, 2.34)
Service/sales	0.58 (0.42, 0.80)	0.83 (0.56, 1.24)	1.38 (0.87, 2.18)	0.74 (0.40, 1.34)		1.05 (0.63, 1.74)
Outdoor/housewife	0.63 (0.37, 1.06)	0.85 (0.45, 1.60)	1.62 (0.87, 3.01)	0.72 (0.31, 1.69)		0.48 (0.22, 1.02)
Cancer patient status						
General public	1.00 (reference)					
Cancer patients or family	1.12 (0.89, 1.40)					
Cancer information sources (multiple responses)						
Medical professionals			1.17 (0.94, 1.47)			1.57 (1.22, 2.03)
Personal	0.73 (0.49, 1.08)		0.66 (0.43, 0.99)	0.57 (0.35, 0.94)		
Books				1.38 (0.97, 1.97)		
Mass media	1.72 (1.39, 2.12)		1.29 (1.04, 1.61)	1.45 (1.08, 1.94)	0.69 (0.53, 0.89)	1.25 (0.97, 1.61)
Internet	1.60 (1.26, 2.02)	2.26 (1.74, 2.92)		1.22 (0.87, 1.72)	1.52 (1.14, 2.02)	1.27 (0.95, 1.71)
Determinant of SNS use						
System quality	1.00 (reference)	1.00 (reference)	1.00 (reference)			
Information quality	0.73 (0.58, 0.92)	1.46 (1.12, 1.92)	0.74 (0.59, 0.94)			
Communication quality	0.80 (0.61, 1.05)	1.81 (1.30, 2.54)	0.85 (0.64, 1.13)			
Source of NCIC SNS awareness						
Recommendation by acquaintance			1.00 (reference)	1.00 (reference)	1.00 (reference)	1.00 (reference)
Other’s SNS			1.76 (1.20, 2.58)	1.38 (0.74, 2.54)	0.51 (0.30, 0.88)	1.20 (0.78, 1.84)
Online searching			1.02 (0.67, 1.56)	0.89 (0.44, 1.79)	0.43 (0.24, 0.78)	0.68 (0.41, 1.10)
Website/blog of the NCIC			2.23 (1.38, 3.60)	2.31 (1.17, 4.58)	0.87 (0.45, 1.69)	1.00 (0.60, 1.68)
Online events hosted by the NCIC			1.41 (0.91, 2.20)	1.75 (0.89, 3.46)	0.61 (0.33, 1.13)	0.72 (0.43, 1.19)
Printed materials			2.03 (1.24, 3.33)	1.05 (0.50, 2.21)	0.50 (0.26, 0.96)	0.75 (0.42, 1.34)
Time of day of SNS use (multiple responses)						
1-5 a.m.		0.52 (0.29, 0.93)				
9 a.m. to 12 p.m.				1.41 (1.06, 1.88)		1.28 (1.01, 1.63)
1-6 p.m.	1.34 (1.09, 1.64)			1.54 (1.15, 2.07)		1.36 (1.05, 1.75)
7-9 p.m.		1.23 (0.96, 1.59)				1.27 (0.99, 1.64)
10 p.m. to 12 a.m.	1.43 (1.11, 1.84)					1.45 (1.08, 1.95)
	R^2^=0.068	R^2^=0.042	R^2^=0.059	R^2^=0.088	R^2^=0.023	R^2^=0.083
	p<0.001	p<0.001	p<0.001	p<0.001	p<0.001	p<0.001

Values are presented as adjusted odds ratio (95% confidence interval). SNS, social networking service; NCIC, National Cancer Information Service.

1 Gender, age, occupation, cancer patient status, cancer information sources (personal, mass media, Internet), determinant of SNS use, and time of day of SNS use (1-6 p.m., 10 p.m. to 12 a.m.) were adjusted for.

2 Gender, age, occupation, cancer information sources (Internet), determinant of SNS use, and time of day of SNS use (1-5 a.m., 7-9 p.m.) were adjusted for.

3 Age, occupation, cancer information sources (medical professionals, personal, mass media), determinant of SNS use, and source of NCIC SNS awareness were adjusted for.

4 Gender, occupation, cancer information source (personal, books, mass media, Internet), source of NCIC SNS awareness, and time of day of SNS use (9 a.m. to 12 p.m., 1-6 p.m.) were adjusted for.

5 Age, cancer information sources (mass media, Internet), and source of NCIC SNS awareness were adjusted for.

6 Age, occupation, cancer information sources (medical professionals, mass media, Internet), source of NCIC SNS awareness, and time of day of SNS use (9 a.m. to 12 p.m., 1-6 p.m., 7-9 p.m., 10 p.m. to 12 a.m.) were adjusted for.

**Table 3.** Factors associated with satisfaction with NCIC SNSs (N=1,601)^1^

	B	t	p-value
Age (yr)			
10-29	Reference		
30-39	-0.06	-2.26	0.02
40-49	-0.01	-0.19	0.84
≥50	0.02	0.73	0.46
Cancer information sources (multiple responses)			
Medical professionals	0.04	1.75	0.08
Personal	-0.02	-0.93	0.35
Internet	0.07	2.78	0.005
Use of Facebook	0.03	1.17	0.24
Determinant of SNS use			
System quality	Reference		
Information quality	0.08	3.12	0.002
Communication quality	0.03	1.33	0.18
Use of NCIC Twitter			
None	Reference		
Passive	0.01	0.10	0.92
Active	0.09	3.42	0.001
Use of NCIC Facebook			
None	Reference		
Passive	0.02	0.15	0.63
Active	0.12	4.44	<0.001
Source of NCIC SNS awareness			
Recommendation from an acquaintance	Reference		
Other SNSs	-0.09	-2.09	0.04
Online searching	-0.08	-2.09	0.04
Website/Blog of the NCIC	-0.02	-0.71	0.47
Online events hosted by the NCIC	-0.06	-1.85	0.06
Printed materials	-0.10	-3.21	0.001
	R^2^ =0.131, p<0.001		

SNS, social networking service; NCIC, National Cancer Information Service.

1 All variables were adjusted using linear regression analysis.
